# Transcriptome Analysis of Rice Near-Isogenic Lines Inoculated with Two Strains of *Xanthomonas oryzae* pv. *oryzae*, AH28 and PXO99^A^

**DOI:** 10.3390/plants13223129

**Published:** 2024-11-07

**Authors:** Pingli Chen, Xing Zhang, Xiaogang Li, Bingrui Sun, Hang Yu, Qing Liu, Liqun Jiang, Xingxue Mao, Jing Zhang, Shuwei Lv, Zhilan Fan, Wei Liu, Wenfeng Chen, Chen Li

**Affiliations:** 1 Rice Research Institute, Guangdong Academy of Agricultural Sciences, Guangzhou 510640, China; 2 Key Laboratory of Genetics and Breeding of High Quality Rice in Southern China (Co-Construction by Ministry and Province), Ministry of Agriculture and Rural Affairs, Guangzhou 510640, China; 3 Guangdong Key Laboratory of New Technology in Rice Breeding, Guangzhou 510640, China; 4 Guangdong Rice Engineering Laboratory, Guangzhou 510640, China; 5 Hanzhong Agricultural Technology Promotion and Training Center, Hanzhong 723000, China

**Keywords:** *Xanthomonas oryzae*, transcriptome, differentially expressed genes, AH28, *Xa23*

## Abstract

Rice bacterial blight (BB), caused by *Xanthomonas oryzae* pv. *oryzae* (*Xoo*), is a major threat to rice production and food security. Exploring new resistance genes and developing varieties with broad-spectrum and high resistance has been a key focus in rice disease resistance research. In a preliminary study, rice cultivar Fan3, exhibiting high resistance to PXO99^A^ and susceptibility to AH28, was developed by enhancing the resistance of Yuehesimiao (YHSM) to BB. This study performed a transcriptome analysis on the leaves of Fan3 and YHSM following inoculation with *Xoo* strains AH28 and PXO99^A^. The analysis revealed significant differential expression of 14,084 genes. Among the transcription factor (TF) families identified, bHLH, WRKY, and ERF were prominent, with notable differences in the expression of *OsWRKY62*, *OsWRKY76*, and *OsbHLH6* across samples. Over 100 genes were directly linked to disease resistance, including nearly 30 NBS–LRR family genes. Additionally, 11 SWEET family protein genes, over 750 protein kinase genes, 63 peroxidase genes, and eight phenylalanine aminolysase genes were detected. Gene ontology (GO) analysis showed significant enrichment in pathways related to defense response to bacteria and oxidative stress response. Kyoto Encyclopedia of Genes and Genomes (KEGG) enrichment analysis indicated that differentially expressed genes (DEGs) were enriched in phenylpropanoid biosynthesis and diterpenoid biosynthesis pathways. Gene expression results from qRT-PCR were consistent with those from RNA-Seq, underscoring the reliability of the findings. Candidate genes identified in this study that may be resistant to BB, such as NBS–LRR family genes *LOC_Os11g11960* and *LOC_Os11g12350*, SWEET family genes *LOC_Os01g50460* and *LOC_Os01g12130*, and protein kinase-expressing genes *LOC_Os01g66860* and *LOC_Os02g57700*, will provide a theoretical basis for further experiments. These results suggest that the immune response of rice to the two strains may be more concentrated in the early stage, and there are more up-regulated genes in the immune response of the high-resistant to PXO99A and medium-resistant to AH28, respectively, compared with the highly susceptible rice. This study offers a foundation for further research on resistance genes and the molecular mechanisms in Fan3 and YHSM.

## 1. Introduction

Rice (*Oryza sativa* L.) is a vital food crop, especially in Asia, playing a crucial role in global agriculture [1]. Bacterial blight (BB), caused by *Xanthomonas oryzae* pv. *oryzae* (*Xoo*), is a major threat to rice production, potentially reducing yields by 20% to 30%, with severe cases leading to up to 50% or even total crop failure [2,3,4,5]. This vascular bundle disease is not effectively managed by chemical treatments, making the cultivation of resistant rice varieties a key strategy for control [3,6,7].

To develop resistant varieties, it is essential to continuously identify stable resistance genes and rice germplasm with these traits. Many avirulence (*avr*) or virulence (*vir*) genes associated with BB encode transcription activator-like effector (TALE) proteins [8,9]. These TALE proteins, secreted by *Xoo* into cell nuclei, bind to effector-binding elements (EBEs) in the promoters of resistance (*R*) or susceptibility (*S*) genes, acting as transcription factors (TFs) that either activate or suppress these genes, thus influencing disease resistance [8,9,10].

Currently, 47 *R* genes of rice BB have been identified [11]. Dominant *R* genes include *Xa1* [12], *Xa7* [13], *Xa3*/*Xa26* [14], *Xa10* [15], *Xa21* [16], *Xa23* [10], and *Xa27* [17], while recessive genes include *xa5* [18], *xa13* [19], *xa41(t)* [20], and *xa44* [21]. These genes encode various proteins: *Xa1* and its allelic R genes, *Xa2*, *Xa31(t)*, *Xa14*, *CGS-Xo111*, and *Xa45(t)* encode nucleotide-binding leucine-rich repeat (NLR) proteins [22]; *Xa3*/*Xa26* and *Xa21* encode receptor-like kinases (RLKs); *Xa4* encodes a cell-wall-associated kinase [23]; *xa5* encodes a TFIIAγ protein; *OsSWEET5* [24], *xa13*, *xa41(t)*, *xa25* [25], *OsSWEET11b* [26], and *OsSWEET12* [27] encode MtN3/saliva/SWEET proteins; *Xa7*, *Xa27*, *Xa10*, and *Xa23* are executor R genes. Most of these genes are TALE-mediated, except RLK genes *Xa3*/*Xa26*, *Xa4*, and *Xa21* [28]. *Xa23*, derived from wild rice in China, provides high resistance to nearly all *Xoo* strains and is widely used in breeding due to its advantages of whole-growth, dominant, and broad-spectrum resistance [29,30]. *Xa23* is an executor *R* gene, activated by binding of *AvrXa23*, a common non-toxic effector in *Xoo*, to induce transcription expression of *Xa23* [10]. It has the same open reading frame (ORF) as the susceptible allele *xa23*, but their promoter region is different, and *Xa23* lacks the TALE binding element with *AvrXa23* [10]. BB pathogens evolve under natural and artificial pressures, leading to potential resistance breakdown. Research by Chen Gongyou et al. found that rice strains with *xa5*, *Xa7*, and *Xa23* had broad-spectrum resistance to most *Xoo* strains, while other genes were more easily overcome [31]. However, strains such as AH28 and P99M2 have breached *Xa23* resistance [31,32]. After inoculating CBB23 with wild PXO99^A^ and its mutant P99M2, 1235 differentially expressed genes (DEGs) were detected by RNA-Seq analysis [32]. The ribosome and biosynthesis of phenylpropanoids in the KEGG pathway were found to be significantly enriched in DEGs. RNA-seq analysis of near-isogenic lines CBB23 and JG30 post-PXO99^A^-infection identified 1645 DEGs related to resistance [33]. Inoculating CBB23 with AH28 and PXO99^A^ revealed 7997 DEGs, including 431 TFs. Most differential metabolites were alkaloids, terpenes, amino acids, and derivatives [34].

Due to the poor persistence of most *R* genes, the search for stable and durable *R* genes is vital for genetic improvement and food security. Yuehesimiao (YHSM), known for disease resistance, high yield, and quality [35], is susceptible to PXO99^A^. Preliminary experiments enhanced YHSM resistance using BB-resistant PR1, producing the highly resistant Fan3 variety. We found that YHSM showed moderate resistance (MR) to AH28, while Fan3 showed susceptibility (S). At present, the candidate genes related to resistance to BB were obtained by transcriptome analysis conducted by inoculating rice susceptible to AH28 and resistant to PXO99A. However, no candidate genes related to BB have been obtained from rice inoculations that are resistant to AH28. This study involved inoculating Fan3 and YHSM seedlings with AH28 and PXO99^A^ and conducting transcriptome analysis to identify potential new *R* genes, laying the groundwork for molecular breeding and *R* gene localization against BB. The candidate genes obtained in this study have been designed by molecular markers of two parents, YHSM and Fan3, and marker-assisted selection has been carried out in breeding.

## 2. Results

### 2.1. Observations of the Susceptibility of Rice Inoculated with Xoo Strains AH28 and PXO99^A^

**Figure 1:**
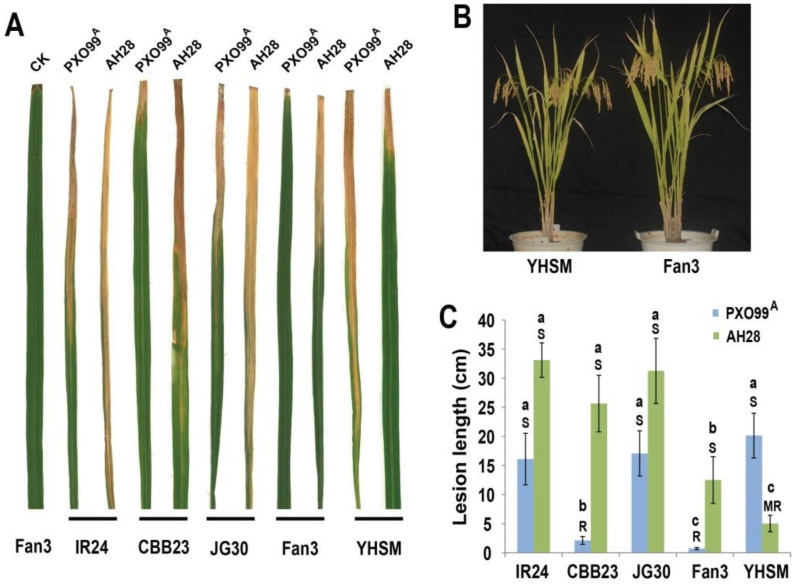
(**A**) Comparison of pathogenicity of two *Xoo* strains in different rice. CK was the control variety of uninfected *Xoo*. IR24 was the control susceptible variety of inoculation, CBB23 and JG30 were near-isogenic lines (NILs), and Fan3 and YHSM were NILs. Only CBB23 and Fan3 contained the *Xa23* gene; IR24 and JG30 did not contain the known resistance genes to BB; YHSM contained *Xa4*, but did not contain resistance genes *xa5*, *Xa7*, *Xa21*, and *Xa23*. (**B**) Plant type comparison of Fan3 and YHSM. (**C**) Lesion length statistics and disease resistance analysis of all varieties inoculated with two strains of BB. Lesion length <3 cm indicated disease resistance (R), 3–6 cm indicated medium resistance (MR), and >10 cm indicated disease sensitivity (S). Values are the mean from 10 biological replicates. Different lowercase letters above the bars and disease resistance in the figures represent significant differences as determined by Duncan’s test at *p* < 0.05.

Five rice varieties (IR24, CBB23, JG30, Fan3, and YHSM) were inoculated with *Xoo* strains AH28 and PXO99^A^ using the clipped leaf method, and lesion lengths were observed and measured. The results indicated significant differences in resistance levels among the varieties against the two strains (Figure 1A). CBB23 and Fan3 exhibited resistance (R) to PXO99^A^, with lesion lengths of 2.12 cm and 0.72 cm, respectively. The remaining three varieties were S to PXO99^A^. For the AH28 strain, only YHSM showed MR, with a lesion length of 5.02 cm, while the other varieties were S, with CBB23 displaying a lesion length of 25.65 cm (Figure 1). Fan3, genetically modified to improve BB resistance in the YHSM background (Figure 1B), demonstrated high resistance to PXO99^A^, showing lesion lengths of 0.72 cm and 20.13 cm for Fan3 and YHSM, respectively, indicating R and S. However, after AH28 inoculation, lesion lengths were 12.50 cm for Fan3 (S) and 5.02 cm for YHSM (MR) (Figure 1C). Fan3 exhibited consistent resistance grades against both strains, but with shorter lesion lengths compared to CBB23. After inoculation with PXO99A, IR24, JG30, and YHSM were all susceptible to PXO99A, and there was no significant difference in lesion length, while CBB23 and Fan3 were resistant to PXO99A, and the differences in lesion length were significant between them and other cultivars. After inoculation with AH28, IR24, CBB23, and JG30 were all susceptible to AH28, and there was no significant difference in lesion length, while Fan3 and YHSM were susceptible and moderately resistant, respectively, and the differences in lesion length were significant between them and other cultivars. The resistance levels of Fan3 and YHSM to the two strains differed significantly; Fan3 showed enhanced resistance to PXO99^A^ but reduced resistance to AH28 compared to YHSM. These findings highlight that the resistance of a single variety can vary between strains, and no variety was resistant to both strains. Apart from YHSM, lesion lengths of other varieties inoculated with AH28 were significantly longer than those inoculated with PXO99^A^.

### 2.2. Transcriptome and DEG Analysis

To investigate the genes associated with BB resistance in Fan3 and YHSM, leaves were inoculated with *Xoo* strains AH28 and PXO99^A^. Leaf samples were collected at 1 h post-inoculation (hpi), 1 day post-inoculation (dpi), and 2 dpi for RNA-seq analysis. For Fan3 inoculated with AH28, the samples were labeled as FAH1, FAH2, and FAH3, while those inoculated with PXO99^A^ were named FNI1, FNI2, and FNI3. For YHSM inoculated with AH28, the samples were named YAH1, YAH2, and YAH3, and for PXO99^A^-inoculated samples, they were YNI1, YNI2, and YNI3.

**Figure 2:**
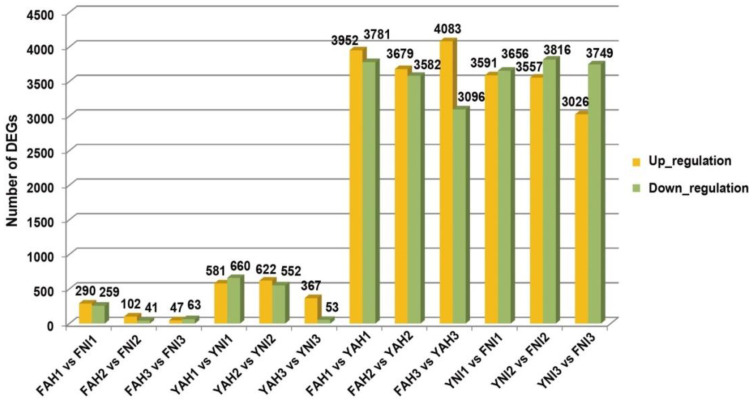
Statistical analysis of significant differentially expressed genes (DEGs) compared between different groups. The orange and green bars indicate up-regulated and down-regulated DEGs, respectively.

A total of about 209.28 GB of clean data was obtained through RNA-Seq, with an average of 5.81 GB per sample. About 149.10 million raw reads were obtained, and 139.52 million raw reads with a total of 33.66% mapping rate were mapped to the transcriptome. The Q30 values exceeded 94.93%, and the mapping efficiency of clean reads to the reference genome ranged from 92.00% to 96.02%. We performed DEGs analysis based on RNA-seq data under a comprehensive comparison of two paired-end read lengths (150 bp). These results confirmed high sequence quality and data reliability, suitable for subsequent bioinformatics analysis. A total of 14,084 DEGs were identified across different strains and inoculation stages (Table S1). The DEGs for each comparison were as follows: FAH1 vs. FNI1 (up-regulated 290, down-regulated 259), FAH2 vs. FNI2 (up-regulated 102, down-regulated 41), FAH3 vs. FNI3 (up-regulated 47, down-regulated 63), YAH1 vs. YNI1 (up-regulated 581, down-regulated 660), YAH2 vs. YNI2 (up-regulated 622, down-regulated 552), and YAH3 vs. YNI3 (up-regulated 367, down-regulated 53) (Figure 2). Furthermore, the number of DEGs between Fan3 and YHSM inoculated with the same strain was analyzed (Figure 2). More DEGs were detected in YHSM than in Fan3. Significant numbers of DEGs were observed when comparing samples infected with the same strain at the same time points (Figure 2).

### 2.3. Analysis of TF Representation in DEGs

**Figure 3:**
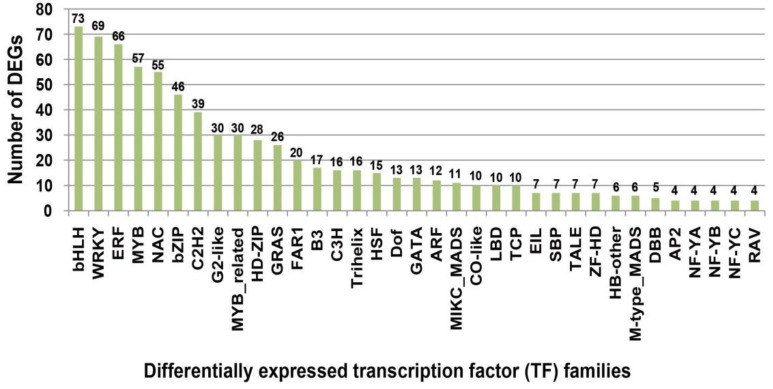
The number of differentially expressed transcription factors (TFs) in response to inoculation by two strains. The figure shows some TF families. Families with less than four DEGs detected are not shown.

**Figure 4:**
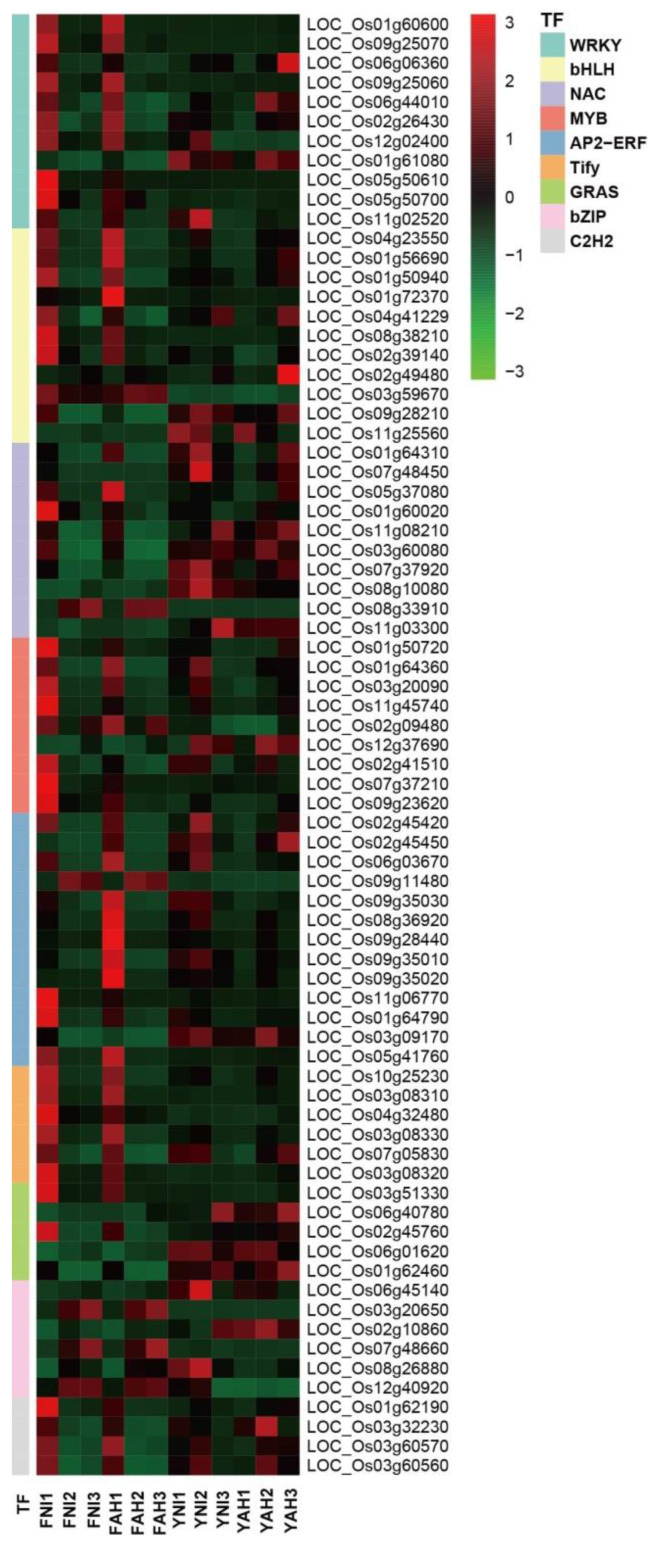
The differential expression of TFs (WRKY, bHLH, NAC, MYB, AP2-ERF, Tify, GRAS, bZIP, and C2H2) in response to the inoculation of two strains. TFs showed increased expression and were more sensitive to immune response in the early stage when the two strains were inoculated with Fan3 compared with YHSM. After the value was homogenized, the color depth indicated the difference between the gene expression and the mean value.

Analysis of DEGs revealed the involvement of numerous TFs in the resistance response post-*Xoo*-inoculation. A total of 778 significantly differentially expressed TFs spanning 51 different families were identified. Notably, MYB, bZIP, WRKY, ERF, and NAC families showed varying degrees of differential expression, with the bHLH TF family, with 73 identified members, being the most prominent (Figure 3 and Figure 4). WRKY and ERF families were also significant, with 69 and 66 identified members, respectively. Examining disease-related TFs within the DEGs, differential expression was observed in 14 WRKY, 6 bHLH, 5 NAC, and 3 MYB TFs. Specifically, WRKY TFs like *OsWRKY62*, *OsWRKY76*, *OsWRKY72*, *OsWRKY71*, *OsWRKY53*, *OsWRKY28*, and *OsWRKY42*, bHLH TFs such as *OsbHLH6*, *OsbHLH10*, and *OsbHLH34*, NAC TFs including *OsNAC4*, and MYB TFs such as *OsMYB21*, *OsMYB30*, and *OsMYB4* were differentially expressed. The expression levels of *OsWRKY62*, *OsWRKY76*, and *OsbHLH6* varied significantly across multiple samples. Uncloned TFs also exhibited significant differential expression, including MYB TFs *LOC_Os01g50720*, *LOC_Os01g64360*, and *LOC_Os05g07010*; ERF TFs *LOC_Os02g45420*, *LOC_Os06g03670*, and *LOC_Os01g04750*; bHLH TFs *LOC_Os01g56690*, *LOC_Os04g41229*, and *LOC_Os08g38210*; and WRKY TFs *LOC_Os06g06360* and *LOC_Os12g02400*. These significantly differentially expressed TFs, detected multiple times, can be further verified in subsequent experiments for their potential roles in BB resistance. Other TFs related to functions like tillering number and drought stress were also identified, but their specific roles in disease resistance require further investigation.

### 2.4. DEGs Closely Related to Disease Resistance

**Figure 5:**
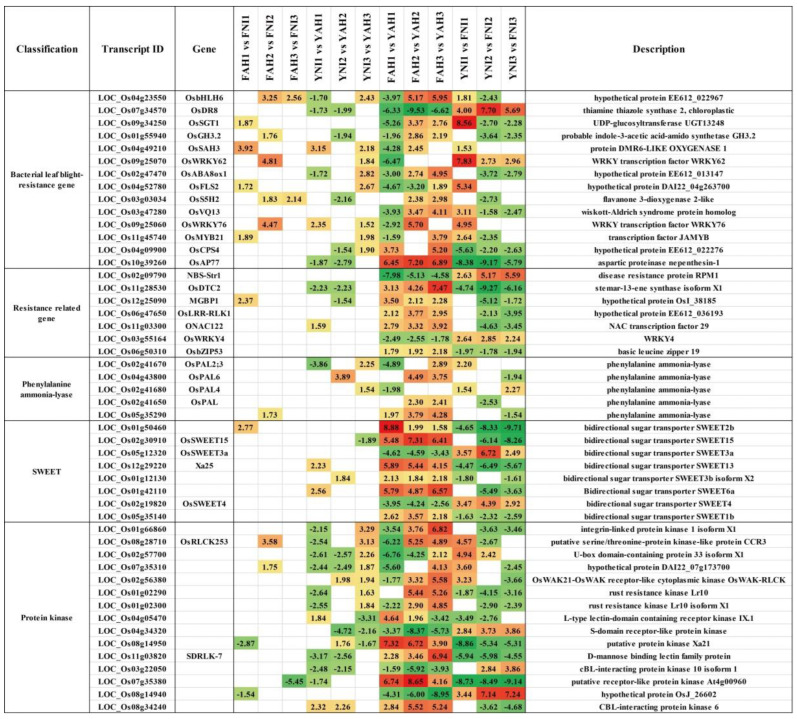
The differential expression of BB gene, resistance-related gene, phenylalanine ammonia-lyase gene, SWEET family gene, and protein kinase among different groups. The DEGs detected in the comparison between different rice inoculated with the same strain increased significantly, and the DEGs detected in the comparison between the same rice inoculated with different strains were significantly decreased. The numbers were the ratios between the comparison groups, with green representing down-regulated, red representing up-regulated, and white representing no significant difference.

Further analysis of DEGs was conducted to identify genes potentially related to BB resistance in rice, focusing on biological processes such as pathogen defense response, signaling, protein synthesis and modification, and metabolism (Figure 5). Over 50 cloned genes directly involved in BB resistance were analyzed, including *OsbHLH6*, *OsDR8*, *OsSGT1*, *OsGH3.2*, and *OsS5H1*, though no differential expression of *Xa23* was detected. Additionally, more than 100 cloned genes related to general disease resistance were found, such as *NBS-Str1*, *OsKSL11*, *OsJAZ12*, *OsPR10*, and *OsPR1a*. Genes directly related to blast resistance included *OsERF104*, *OsWRKY62*, *OsACS2*, and *OsIRL*, while those related to sheath blight included *OsPAL1*, *OsWRKY53*, *OsWRKY30*, and *OsWRKY13*. The NBS–LRR family, critical for plant disease resistance and immunity, was well-represented, with nearly 30 members detected, including *LOC_Os11g11960*, *LOC_Os11g12350*, and *LOC_Os11g11770*. SWEET sugar transporters, targeted by extracellular pathogens for sugar nutrient acquisition, were also prominent, with 11 potential SWEET family genes identified, such as *OsSWEET15*, *OsSWEET3a*, *Xa25*, and *OsSWEET4*, along with seven uncloned genes: *LOC_Os01g50460*, *LOC_Os01g12130*, *LOC_Os01g42110*, *LOC_Os01g65880*, *LOC_Os05g35140*, *LOC_Os01g36070*, and *LOC_Os03g22200*. Additionally, more than 750 protein-kinase-expressing genes were detected, including *OsFLS2*, *BSR1*, *OsSAPK9*, and *OsCPK4*, and uncloned genes *LOC_Os01g66860*, *LOC_Os02g57700*, and *LOC_Os07g35310*. Differential expression was observed in 16 cloned genes related to lignin anabolism, many of which are functionally verified for rice disease resistance. Also identified were 63 peroxidase (POD), eight phenylalanine ammonia lyase (PAL), two cinnamate-4-hydroxylase (C4H), three cinnamyl alcohol dehydrogenase (CAD), and 24 chitinase (CHI) protein-coding genes, including cloned and disease-resistant related genes *POX22.3*, *POXN*, *OsPAL6*, *OsPAL4*, and *OsPAL*.

Hormones regulating plant growth and disease resistance were also examined, identifying DEGs related to hormone metabolism, including *OsOPR1*, *OsDR10*, *OsAP77*, *OsPAL6*, and *ONAC131*. Furthermore, DEGs encoding calmodulin-binding protein, metallothionein, calcium-transporting ATPase, terpene synthetase, and glycosyltransferase, which are associated with plant disease resistance, were detected (Figure 5).

### 2.5. Gene Ontology (GO) Function Analysis and Kyoto Encyclopedia of Genes and Genomes (KEGG) Pathway Analysis

**Figure 6:**
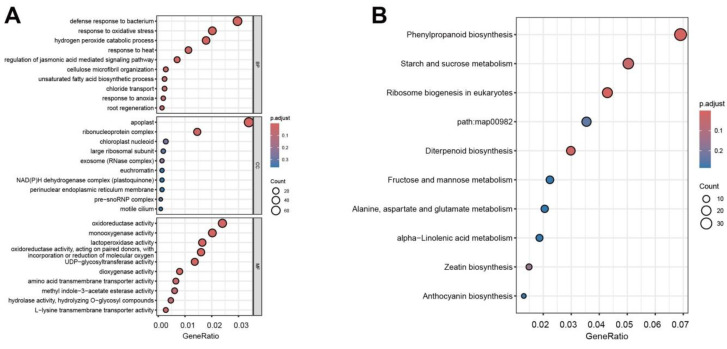
The GO analysis (**A**) of biological process (BP), cellular component (CC), and molecular function (MF), and KEGG pathways analysis (**B**) showing DEGs of six groups (FAH1 vs. FNI1, FAH2 vs. FNI2, FAH3 vs. FNI3, YNI1 vs. YAH1, YNI2 vs. YAH2, and YNI3 vs. YAH3) with clustering of different color patterns. The horizontal coordinate is GeneRatio, that is, the proportion of the genes in the entry to all DEGs, and the vertical coordinate is each GO annotation or KEGG pathway. The size of the dots represents the number of DEGs annotated in the pathway, and the color of the dots represents the *p* adjustment. Different-colored boxes show the level of significance of each GO term and KEGG pathway.

To clarify the functional roles of DEGs in Fan3 and YHSM, GO functional enrichment and KEGG signaling pathway analysis were performed. Six groups (FAH1 vs. FNI1, FAH2 vs. FNI2, FAH3 vs. FNI3, YNI1 vs. YAH1, YNI2 vs. YAH2, and YNI3 vs. YAH3) were analyzed to account for differences in genetic backgrounds between Fan3 and YHSM. GO analysis categorized DEGs into three main categories: cellular component (CC), biological process (BP), and molecular function (MF). A total of 1670 DEGs matched at least one GO term and were further refined into 1028 significantly enriched functional groups, 571 BP, 86 CC, and 371 MF (Figure 6A). In BP, 63 DEGs were mainly enriched in defense response to bacterium (GO:0042742), 43 in response to oxidative stress (GO:0006979), 43 in protein ubiquitination (GO:0016567), and 38 in hydrogen peroxide catabolic process (GO:0042744). Significant enrichment was observed in the CC terms apoplast (GO:0048046) and ribonucleoprotein complex (GO:1990904). In MF, 51 DEGs were significantly enriched in oxidoreductase activity (GO:0016491), 35 in lactoperoxidase activity (GO:0140825), and 29 DEGs in UDP-glycosyltransferase activity (GO:0008194). To determine which MFs are regulated differently to resist pathogen invasion, we performed a GO analysis of the DEGs identified in the six groups (Figure 6A). These results suggest that some disease-resistant proteins or enzymes are involved in transcription, translation, and modification, potentially regulating the biosynthesis and metabolism of secondary metabolites to resist pathogen invasion.

**Figure 7:**
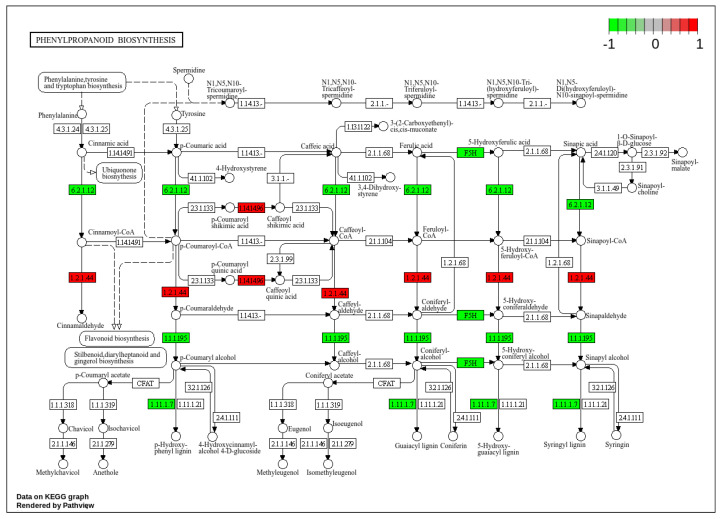
Based on the log_2_FC value of DEGs in YNI1 vs. YAH1, a hypothesized phenylpropanoid biosynthesis pathway was constructed. The numbers in each box represent the code of the enzyme. The green and red boxes indicated down-regulated and up-regulated genes, respectively.

KEGG pathways analysis annotated 1600 genes, matching them to 536 pathways. The main enriched pathways in the six groups included phenylpropanoid biosynthesis, starch and sucrose metabolism, ribosome biogenesis in eukaryotes, diterpenoid biosynthesis, fructose and mannose metabolism, alanine, aspartate, and glutamate metabolism, and anthocyanin biosynthesis (Figure 6B). Significant enrichment was found in phenylpropanoid biosynthesis, diterpenoid biosynthesis, ribosome biogenesis in eukaryotes, and starch and sucrose metabolism pathways. The phenylpropanoid biosynthesis pathway was used to reveal the most relevant biological functions of DEGs in YNI1 vs. YAH1 (Figure 7). Significantly elevated DEGs included 5-O-(4-coumaroyl)-D-quinate 3′-Monooxygenase (EC:1.14.14.96) and cinnamoyl-CoA reductase (EC:1.2.1.44), while DEGs from 4-coumarate-CoA ligase (EC:6.2.1.12), ferulate 5-hydroxylase (EC:1.14.-.-), cinnamyl-alcohol dehydrogenase (EC:1.1.1.195), and peroxidase (EC:1.11.1.7) were significantly down-regulated. These results indicate that pathways involved in *Xoo* in Fan3 and YHSM were mainly concentrated in phenylpropanoid biosynthesis, ribosome biogenesis in eukaryotes, and various metabolite synthesis pathways.

### 2.6. Data Validation via Quantitative Reverse Transcriptase PCR (qRT-PCR)

**Figure 8:**
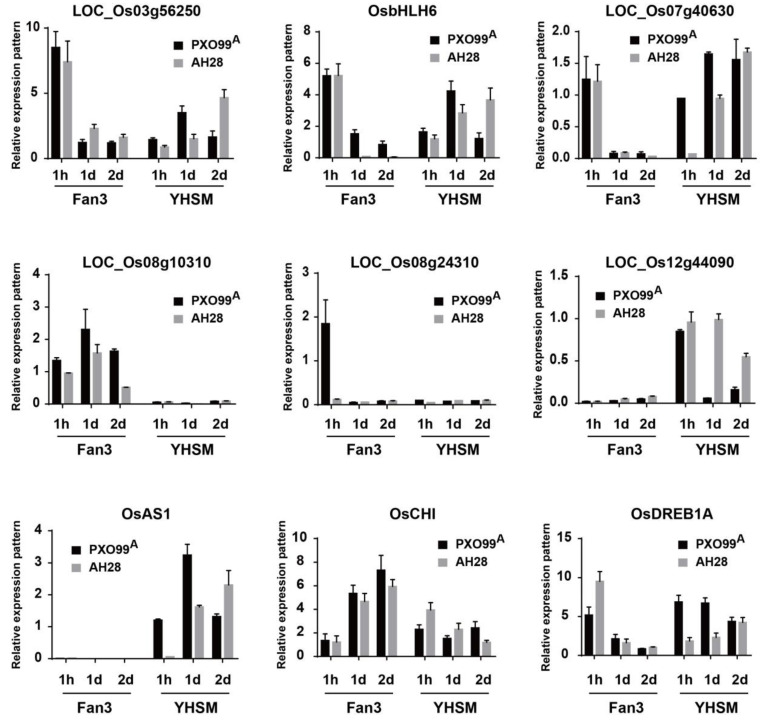
The responses of DEGs to AH28 and PXO99^A^ at different times were verified by qRT-PCR. DEGs were randomly selected for qRT-PCR. The values were mean ± SD of the three biological replicates. 1 h, 1 d, and 2 d refer to 1 h after inoculation (hpi), 1 d after inoculation (dpi), and 2 dpi, respectively.

To verify the reliability of RNA-Seq data, nine DEGs were randomly selected for qRT-PCR validation (Figure 8). These DEGs include genes *OsbHLH6*, *OsAS1*, *OsCHI*, and *OsDREB1A*, and other DEGs, such as *LOC_Os03g56250*, *LOC_Os07g40630*, *LOC_Os08g10310*, *LOC_Os08g24310*, and *LOC_Os12g44090*. The *EF1α* gene served as the internal control gene during qRT-PCR. As shown in Figure 8, the relative expression levels of the nine DEGs were consistent with the expression trends observed in RNA-seq results, demonstrating the reliability of the RNA-seq data.

## 3. Discussion

### 3.1. Potential Resistance Genes Identified in This Study

To resist inoculation by *Xoo*, host plants co-evolved to produce *R* genes. Resistance to BB is regulated by resistance genes, signal transducers, and other stress-related genes. Genes directly and indirectly participating in rice resistance to BB have been cloned, including those encoding NBS–LRR, SWEET family proteins, protein kinases, and TFs [36,37,38,39]. The gene *Xa1* is the first cloned NLR-type *R* gene in rice [12], and it was not until 2016 that it was discovered that all typical TALE proteins activate *Xa1*. According to this discovery, the *Xa1* alleles *Xa2*, *Xa14*, *Xa31(t)*, and *Xa45(t)* were subsequently cloned [22]. RLKs play an important role in the transmission of plant immune signals in response to pathogen infection and have been widely utilized in rice disease resistance breeding [40,41]. Disease-resistant substances (lignin, flavonoids, and total phenols) and enzymes such as POD, PAL, C4H, 4-coumarate CoA ligase (4-CL), CAD, CHI, and β-1,3-glucanases all play crucial roles in rice disease resistance [42,43,44]. Plant hormones such as salicylic acid, jasmonic acid, ethylene, auxin, cytokinin, and brassinolide are widely reported to be involved in plant disease immune responses [45]. In this study, a large number of genes related to disease resistance were detected (Figure 2, Figure 3, Figure 4 and Figure 6), and many disease resistance genes were reported to participate in the immune response to multiple diseases.

SWEET proteins are a class of sugar transporters that can cause sugar leakage from inoculated cells, making plants susceptible to pathogens [46]. These proteins play a key role in plant development and stress response. Currently, the cloned SWEET family genes in rice include *Xa13*/*OsSWEET11*, *Xa25*/*OsSWEET13*, and *Xa41(t)*/*OsSWEET14*. Their corresponding recessive genes, *xa13*, *xa25*, and *xa41(t)*, evade regulation by pathogenic bacteria due to promoter mutations, becoming special recessive disease resistance genes [19,20,25]. In this study, 11 SWEET family genes were detected, including cloned genes *OsSWEET15* and *OsSWEET13* involved in the immune response to BB, and *OsSWEET3a* involved in the immune response to rice sheath blight (Figure 5). Additionally, uncloned genes such as *LOC_Os01g50460*, *LOC_Os01g12130*, *LOC_Os01g42110*, *LOC_Os01g65880*, *LOC_Os05g35140*, and *LOC_Os01g36070* were detected (Figure 5). Among them, *LOC_Os01g50460* showed significantly different expression levels in multiple groups, suggesting its potential involvement in the immune response to BB, warranting further functional validation.

A large number of studies have shown that WRKY, NAC, bHLH, MYB, and other TFs play roles in regulating plant biological and abiotic stress, growth and development, material metabolism, and oxidative senescence [47]. The EBE recognized by PthXo7 in the promoter of the TF *OsTFIIAγ1* gene increases rice disease susceptibility following induction, and *OsTFIIAγ1* can compensate for the defects of *xa5*, thus also being known as an *S* gene [18]. In our study, WRKY TFs were the most frequently detected disease-related TFs, with 69 detected, including *OsWRKY62*, *OsWRKY76*, *OsWRKY42*, *OsWRKY71*, and *OsWRKY53* (Figure 3 and Figure 4). Furthermore, many TFs exhibited significant differential expression in different varieties and strains during induction, demonstrating that TFs play important roles in resistance and sensitivity to BB.

The total amount of DEGs after inoculating Fan3 with two strains was significantly less than that of YHSM, indicating that the genes involved in rice immune response in Fan3 inoculated with two strains are fewer than those in YHSM. Among the three sampling times, at 1 hpi and 1 dpi, the up-regulated genes of FAH vs. FNI were significantly more numerous than down-regulated genes. However, among the three sampling times, only at 1 hpi were there more down-regulated genes of YAH vs. YNI than up-regulated genes. It can also be considered that only at 1 hpi were there more up-regulated genes of YNI vs. YAH than down-regulated genes. When inoculated with the two strains, more DEGs were detected in both cultivars in the early stage. These may also indicate that the immune responses of the cultivars to the two strains were more concentrated in the early stage.

We performed KEGG pathway analysis of DEGs for the immune response of the two strains (Figure 6 and Figure S1). It was found that where Fan3 was inoculated with two strains, DEGs were mainly concentrated in plant hormone signal transduction, MAPK signaling pathway-plant, phenylpropanoid biosynthesis, alpha-linolenic acid metabolism, and other pathways, while for YHSM inoculated with two strains, DEGs were mainly concentrated in diterpenoid biosynthesis, phenylpropanoid biosynthesis, ribosome, ribosome biogenesis in eukaryotes, and other pathways. The KEGG pathways were very different when the two strains were infected with the two cultivars respectively. Only the phenylpropanoid biosynthesis pathway was significantly enriched in the two cultivars. Introducing R gene from PR1 to Fan 3 enhanced the disease resistance to PXO99A, but weakened the disease resistance to AH28. While Fan3 was highly resistant to PXO99A and YHSM was medium-resistant to AH28, most of the enriched KEGG pathways were different, indicating that the immune responses of the two cultivars to the two strains were quite different.

After the two cultivars were infected with AH28, the number of up-regulated genes of FAH vs. YAH was significantly more than that of down-regulated genes. On the other hand, the number of down-regulated genes of YNI vs. FNI was significantly higher than that of up-regulated genes after inoculation with PXO99A. YHSM showed MR to AH28, while Fan3 showed S. This indicated that the expression patterns of the two strains were quite different in the two cultivars. In the study, 14,084 DEGs were detected after near-isogenic lines of YHSM and Fan3 were infected with two strains. Due to the large amount of DEGs detected, only some of DEGs, TFs, and possibly related resistance candidate genes were analyzed in this study. In Figure 2, we can see that after infection of two strains of the same material, the number of up-regulated genes was more than that of down-up-regulated genes. We performed GO and KEGG pathway analysis on the six groups of detected DEGs. We found that the up-regulated and down-regulated genes were detected in a hypothesized phenylpropanoid biosynthesis pathway, and the results of other pathways were also detected in both up-regulated and down-regulated genes (results not shown). However, we were unable to correlate the two cultivars with disease resistance.

### 3.2. Genetic Improvement of BB Resistance

Food security remains a major problem facing human society, with plant diseases causing more than 10% of crop losses worldwide annually, even under controlled conditions [48]. Disease resistance breeding is the most environmentally friendly and effective control measure. However, under the dual pressure of natural and artificial selection, pathogenic bacteria are constantly mutating, making it difficult to breed rice varieties with persistent broad-spectrum resistance to BB through conventional methods. Genes such as *Xa4*, *Xa7*, and *Xa21* have been widely employed in rice breeding for disease resistance, but the large-scale and long-term application of a single resistance gene in cultivated varieties leads to pathogen mutation, ultimately reducing or even nullifying the resistance of these rice varieties [49]. It has been reported that the resistance of *Xa4*, *Xa13*, and *Xa21* can be overcome by most strains [49,50]. Currently, PXO99^A^ has become the dominant *Xoo* in China, overcoming *xa5* resistance, and rice varieties resistant to *xa5* are susceptible to PXO99^A^ [51]. The recessive gene *xa13* and the dominant gene *Xa23* are resistant to PXO99^A^, with *Xa23* being commonly used to enhance rice resistance to PXO99^A^ [10,31,52]. However, *Xa23* resistance has been reported to be overcome by two strains lacking *avrXa23*, one of which is AH28, featured in this study [4,31,32]. The rice genes resistant to the AH28 strain are *xa5*, *Xa7*, and *Xa21* [4]. Since the activation of *R* genes requires matching *avr* genes, pyramiding multiple *R* genes in a single rice variety can effectively reduce the risk of resistance being overcome by some strains [49,50].

The resistance of YHSM was genetically improved by screening the new resistance germplasm PR1, resulting in Fan3, which is resistant to PXO99^A^ and susceptible to AH28. Fan3 showed a significantly reduced lesion length when inoculated with PXO99^A^, while lesion length significantly increased when inoculated with AH28 compared to YHSM (Figure 1). At the same time, the lesion length of Fan3 after inoculation with AH28 and PXO99^A^ was shorter compared to CBB23, which contains only *Xa23* (Figure 1). Under the same genetic background, the DEGs detected by different strains were much fewer than those detected by the same strains under different genetic backgrounds (Figure 2). These results suggest that a large number of genes from PR1 may be involved in the process of improving BB resistance in YHSM, and the genetic background difference between Fan3 and YHSM leads to the detection of many DEGs. Additionally, DEGs detected from Fan3 and YHSM differed after inoculation. After substantial changes in the early stages of inoculation, the number of DEGs of 2 dpi decreased significantly (Figure 2), which may be an effective signal of the rice defense system sensing pathogen inoculation, thus causing subsequent minor transcriptional changes. YHSM contains *Xa4* but lacks *xa5*, *Xa7*, *Xa21*, and *Xa23*, yet it has some resistance to AH28, indicating that YHSM may contain other *R* genes that can resist AH28. This also suggests the possibility of obtaining genetically modified YHSM resistant to both AH28 and PXO99^A^ through polygene polymerization. It is necessary to evaluate and rationally utilize known *R* genes, continuously select new germplasm resources, explore and identify new broad-spectrum *R* genes, breed varieties with persistent resistance, and ensure high and stable yield and quality of rice.

## 4. Conclusions

In this study, Fan3 and YHSM were inoculated with two *Xoo* strains, AH28 and PXO99^A^, and numerous DEGs were detected through transcriptome analysis. Comparative analysis of different varieties and different strains revealed 14,084 DEGs, including more than 100 cloned genes related to disease resistance. Additionally, DEGs encoding NBS–LRR, SWEET family proteins, protein kinases, TFs, and phenylalanine proteins involved in rice resistance to BB were statistically analyzed, showing that many DEGs encode different types of proteins. This study demonstrates the potential to obtain genetically modified YHSM resistant to both AH28 and PXO99^A^ through genetic modification and polymerization. It also provides a foundation for further research on *R* genes and the molecular mechanisms of Fan3 and YHSM.

## 5. Materials and Methods

### 5.1. Plant Materials and Xoo Strains

The rice materials used in this study included IR24, CBB23, JG30, YHSM, and Fan3. Rice CBB23 contained *Xa23*, which is resistant to PXO99^A^. IR24 and JG30 are rice varieties with high susceptibility to *Xoo*. YHSM contained *Xa4* and was bred by the Rice Research Institute of Guangdong Academy of Agricultural Sciences [35]. Fan3 contained *Xa23*, a genetically modified variety based on YHSM, and a new resistance germplasm PR1 from our laboratory. Fan3 had broad-spectrum and high resistance to *Xoo*. All materials were grown in the greenhouse of the Rice Research Institute of Guangdong Academy of Agricultural Sciences, under conventional water and fertilizer management.

The *Xoo* strain AH28 was provided by Professor Gongyou Chen from Shanghai Jiao Tong University [4]. This strain could overcome the resistance of *Xa23*. PXO99^A^ was a model strain inoculated into rice containing *Xa23*, producing a resistance response. All strains were stored in glycerol at −80 °C. Before inoculation, the strains were rejuvenated with nutrient broth medium and cultured at 28 °C for 48 h. Inoculated *Xoo* solution was prepared using sterile water, and the concentration was adjusted to OD_600_ = 1.0.

### 5.2. Inoculation of Two Xoo Strains and Disease Investigation

The scissors-dipping method was used to inoculate seedlings at the five–six-leaf stage [53]. Two newly grown, fully unfolded leaves per plant were inoculated, and samples were collected at 1 hpi, and 1 and 2 dpi. Each of the three biological replicates consisted of at least 15 leaves, which were flash-frozen in liquid nitrogen and stored at −80 °C or sent directly for RNA-seq. Lesion lengths were measured at 14–20 dpi when the disease symptoms stabilized. Lesion lengths were categorized as follows: <3 cm disease resistance (R), 3–6 cm moderate resistance (MR), 6–9 cm moderate sensitivity (MS), and >9 cm disease sensitivity (S) [54].

### 5.3. Transcriptome Library Construction and Sequence

Total RNA was extracted from samples using TRIzol (Invitrogen, Carlsbad, CA, USA). RNA was treated with DNaseI (Takara Bio Inc., Beijing, China) to remove DNA contaminants and then purified using an RNA purification kit (Tiangen Bio. Co., Beijing, China). RNA concentration and quality were measured with an ND-2000 spectrophotometer (Thermo Fisher Scientific Inc., Waltham, MA, USA), and RNA integrity was assessed with an Agilent 2100 (Agilent, Santa Clara, CA, USA).

After quality checks, libraries were constructed. Eukaryotic mRNA was enriched using Oligo(dT) magnetic beads and fragmented using fragmentation buffer. First-strand cDNA was synthesized by 6-base random primers. The purified double-stranded cDNA was synthesized from mRNA using random hexamer primers, followed by second-strand synthesis. The double-stranded cDNA was then end-repaired, A-tailed, and ligated with sequencing adapters. Size selection was performed, and the cDNA library was enriched by PCR. The library’s effective concentration (>2 nmol/L) was quantified by qRT-PCR. Finally, sequencing was performed using Illumina HiSeq.

### 5.4. RNA-Seq and DEG Analysis

Following quality assessment and removal of low-quality sequences and splicing artifacts, transcripts were reconstructed, and gene expression levels were quantified using StringTie. Original gene expression levels were determined by read count values, while fragments per kilobase of transcript per million mapped reads (FPKM) standardized expression levels. DESeq2 software (version R4.1.2) was employed to identify DEGs between sample groups, with the Benjamini–Hochberg method used for *p*-value correction to determine the false discovery rate (FDR). DEGs were filtered based on |log_2_FoldChange| ≥ 1.5 and adjusted *p*-value (*p*adj) ≤ 0.01.

### 5.5. Visual Analysis of Transcriptome Data

RNA-Seq data were analyzed using HiSat2 (version 2.2.1) and HTSeq-count (version 2.0.5) along with DESeq2 (version 1.42.1) to elucidate differentially expressed genes. DEGS were annotated using the InterPro database (version 98.0) (https://www.ebi.ac.uk/interpro/ (accessed on 3 July 2024)) for functional insights and the ITAK database (version 18.12) (http://itak.feilab.net/cgi-bin/itak/index.cgi (accessed on 3 June 2024)) for TF prediction. GO function enrichment analysis was performed with AgriGO software (version 2.0), elucidating the main biological functions of DEGs. KOBAS software (version 109.1) was utilized to analyze KEGG pathway enrichment (https://www.kegg.jp/kegg/ (accessed on 4 June 2024)) to uncover molecular interaction networks with metabolic pathways, with significance defined by *p*-value < 0.05. The IBM SPSS statistics software (version 22.0) was used for one-way analysis of variance (ANOVA) and Duncan multiple comparisons.

### 5.6. Validation of DEGs by RT-PCR and qRT-PCR

To validate RNA-seq findings, nine DEGs were randomly selected for qRT-PCR verification. EF1α served as the internal control. Primers (Table S1) were designed using NCBI Primer-BLAST (https://www.ncbi.nlm.nih.gov/tools/primer-blast/ (accessed on 22 June 2024)). RNA was extracted using TRIzol (Invitrogen, Carlsbad, CA, USA), and cDNA synthesis was performed using a PrimerScript™ RT Reagent Kit (Takara Bio Inc., Kusatsu, Japan). The obtained cDNA was diluted 20 times for qPCR analysis, and the process was repeated in triplicate for each sample, using a SYBR Premix Extaq™ kit (Takara Bio Inc., Kusatsu, Japan), following the manufacturer’s protocols. Relative gene expression levels were analyzed using the comparative CT method (2^−△△CT^) [55].

## Data Availability

The accession number of the original transcriptomic data on NCBI Sequence Read Archive is PRJNA1178446.

## Supplementary Materials

The following supporting information can be downloaded at: https://www.mdpi.com/article/10.3390/plants13223129/s1, Figure S1: The KEGG pathways analysis showing DEGs of six groups: FAH1 vs. FNI1 (A), FAH2 vs. FNI2 (B), FAH3 vs. FNI3 (C), YNI1 vs. YAH1 (D), YNI2 vs. YAH2 (E), and YNI3 vs. YAH3 (F), clustering of different color patterns. The horizontal coordinate is GeneRatio, that is, the proportion of the genes in the entry to all DEGs, and the vertical coordinate is each GO annotation or KEGG pathway. The size of the dots represents the number of DEGs annotated in the pathway, and the color of the dots represents the p adjust. Different colored boxes showed the level of significance of each GO term and KEGG pathway. Table S1: Primer pairs used for qPCR.
