# Supplementary material for: Transcriptome Analysis of Rice Near-Isogenic Lines Inoculated with Two Strains of Xanthomonas oryzae pv. oryzae, AH28 and PXO99A
Source: Plants (Basel). 2024 Nov 7;13(22):3129. doi: 10.3390/plants13223129 (PMC11597379; doi:10.3390/plants13223129)

Supporting information

Figure S1. The KEGG pathways analysis showing DEGs of six groups: FAH1 vs. FNI1 (A), FAH2 vs. FNI2 (B), FAH3 vs. FNI3 (C), YNI1 vs. YAH1 (D), YNI2 vs. YAH2 (E), and YNI3 vs. YAH3 (F), clustering of different color patterns. The horizontal coordinate is GeneRatio, that is, the proportion of the genes in the entry to all DEGs, and the vertical coordinate is each GO annotation or KEGG pathway. The size of the dots represents the number of DEGs annotated in the pathway, and the color of the dots represents the p adjust. Different colored boxes showed the level of significance of each GO term and KEGG pathway.

Figure S1

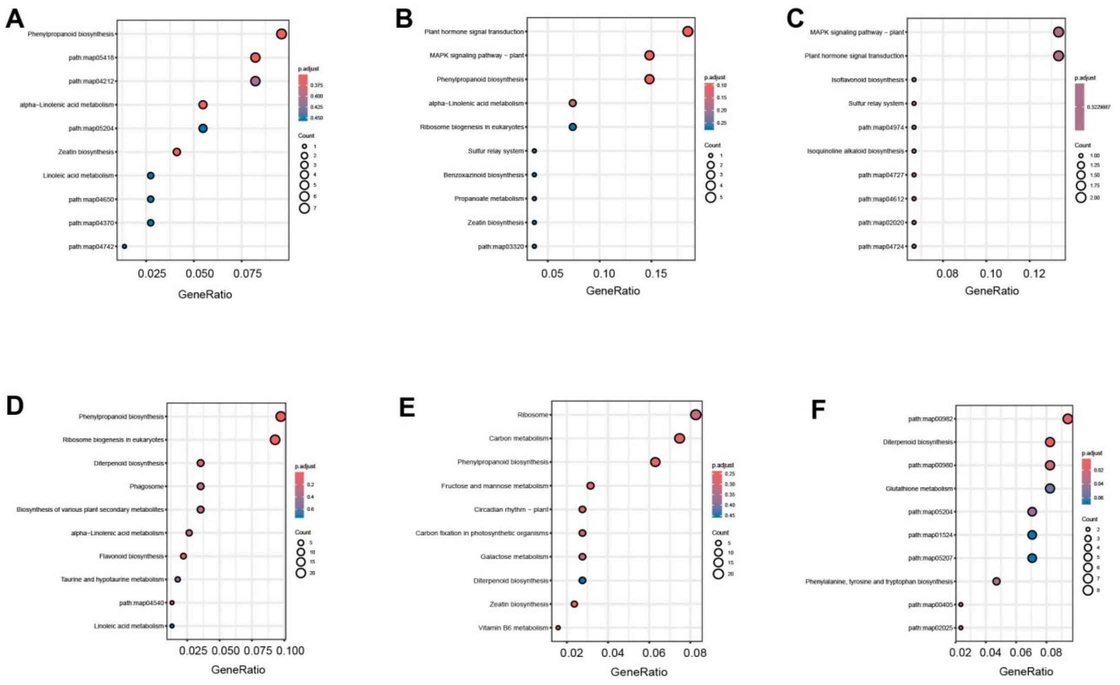

Supplement: Supplementary file 1 [file plants-13-03129-s001.zip › Supporting information.pdf]
